# The Cost of Living with Inherited Ataxia in Ireland

**DOI:** 10.1007/s12311-021-01271-6

**Published:** 2021-07-06

**Authors:** Mark J. Kelly, Petya Bogdanova-Mihaylova, Joshua Skeens, Sharon Moran, Sorcha Farrelly, Richard A. Walsh, Sinéad M. Murphy

**Affiliations:** 1 Trinity College, School of Medicine, Trinity Centre for Health Sciences, Tallaght University Hospital, Dublin, Ireland; 2 Department of Neurology, Tallaght University Hospital, Dublin, Ireland; 3 Academic Unit of Neurology, Trinity College Dublin, Dublin, Ireland

**Keywords:** Ataxia, Inherited ataxia, Friedreich’s ataxia, Spinocerebellar ataxia, Cost of illness, Ireland

## Abstract

Inherited ataxias are a heterogenous group of neurodegenerative disorders characterised by progressive impairment of balance and coordination, typically leading to permanent and progressive disability. Diagnosis and management of these disorders incurs a range of direct and indirect financial costs. The aim of this study was to collect individual ataxia-related healthcare resources in a large cohort of individuals with different subtypes of inherited ataxia and calculate the associated cost of illness in the Republic of Ireland. One hundred twenty-nine respondents completed a cross-sectional study on healthcare resource utilisation for progressive ataxia in Ireland. Costs were calculated using a prevalence-based approach and bottom-up methodology. The COI for inherited ataxia in 2016 was €59,993 per person per year. Results were similar between participants with Friedreich’s ataxia (FRDA, *n* = 56), non-FRDA (*n* = 18) and those with undetermined ataxia (*n* = 55). Indirect costs, based on productivity losses by participants or caregivers, accounted for 52% of the cost of illness. Inherited ataxia is associated with significant health and social care costs. Further funding for inherited ataxia to ease the financial burden on patients, caregivers and healthcare system and improve standards of care compliance is warranted.

## Introduction

The inherited ataxias are rare, clinically and genetically heterogeneous neurodegenerative disorders, characterised by slowly progressive gait instability, often in combination with additional neurological and non-neurological clinical features. These features often necessitate extensive personal and medical care.

In Ireland, a small number of specialist neurologists manage the majority of people with inherited ataxia. Provision of disease monitoring and clinical care can therefore be challenging. Friedreich’s ataxia (FRDA), the most common inherited ataxia [1], has an estimated prevalence of 1/20,000–1/50,000 amongst Caucasians and Ireland has one of the highest estimated prevalence rates in Europe at 1/23,000 [2]. Despite this, no studies to date have assessed healthcare costs or resource utilisation of inherited ataxia in Ireland. Such studies have been carried out in other countries, primarily assessing cost of illness (COI) in FRDA [3, 4] or other ataxia subtypes [5], but little is known about the economic consequences of inherited ataxia as a whole.

Establishing an inherited ataxia diagnosis is often challenging and requires comprehensive clinical assessment and investigations. These include magnetic resonance imaging (MRI), basic and advanced genetic testing and other investigations as clinically indicated (nerve-conduction studies, electromyography, echocardiography, optical coherence tomography, muscle and/or nerve biopsy) [6]. These investigations and the associated clinic visits incur substantial costs.

Following initial presentation and diagnostic evaluation, the disease typically progresses and costs continue to grow. Patients require regular specialist review and investigations such as blood tests and echocardiography. Input from other healthcare professionals (OHPs) including physiotherapy (PT), occupational therapy (OT), speech and language therapy (SALT) is commonplace. Physical disability results in high personal care requirements and many patients ultimately become wheelchair-bound. Associated comorbidities such as diabetes, cardiomyopathy and scoliosis require care under various medical and surgical specialties and patients are at high risk of hospital admission due to acute illness.

The appropriate care of any person with inherited ataxia therefore places a significant burden on health and social care systems, as well as the quality of life of patients and caregivers. This burden translates into financial costs, both direct (due to necessary utilisation of services) and indirect (productivity losses due to patients and caregivers being unable to work).

The aim of this study is to estimate the COI of inherited ataxia in the Republic of Ireland by collecting real-life data from a large cohort of patients on their utilisation of healthcare resources. These results are intended to direct management strategies aimed towards the appropriate allocation of resources and minimising of financial burden on the healthcare system and individuals with this chronic progressive condition.

## Methods

### Participants

This cross-sectional observational study was conducted in 2016. Participants were recruited in the National Ataxia Clinic at Tallaght University Hospital, through surveys posted to Ataxia Ireland members (a charitable organisation for people with inherited ataxia) or distributed at patient meetings nationwide and through a link available via the Ataxia Ireland website. Completion of the survey could be assisted by another individual, where appropriate. Participants of any age with suspected or confirmed genetic ataxia were included. Those with acquired forms of ataxia were excluded. Participants were assigned to one of the three subgroups: FRDA, Non-FRDA (for those who reported confirmed genetic diagnosis different from FRDA) and Unknown (for those without or unaware of a genetically confirmed subtype).

### Survey Design

The survey was designed and tested in a pilot study (*n* = 5). Modifications were made on the basis of participant feedback to improve survey’s ease of use.

Participants completed a questionnaire involving questions on sociodemographic data, details of illness (ataxia type, age at symptom onset and diagnosis, wheelchair use), utilisation of services and claiming of financial supports. Illness-related absences from work were assessed using the Work Productivity and Activity Impairment questionnaire (WPAI) [7].

The recall period was 12 months, apart from the frequency of clinical investigations and the use of assisted transport, which were for a period of 6 months. The recall period for the WPAI was 7 days.

Frequency of service use was recorded in pre-defined frequency options (e.g. once, two to three times, weekly).

### Unit Costs

The unit costs were estimated through the sources denoted in Table 1.

**Table 1:** Sources of unit costs

Cost Unit	Source
GP visits	HSE Statistical Analysis of Claims and Payments 2016 [7]
Outpatient clinic visits	HSE Outpatient Ready Reckoner 2012 National Casemix programme [8]
ED visits	HSE Outpatient Ready Reckoner 2012 National Casemix programme [8]
Inpatient stays	Finance department, TUH
OHP visits	HSE PCRS January 2016 revised consolidated payscales [9]
Investigations	• HIPE healthcare pricing office, finance department, TUH • Clinical chemistry department, TUH • Stubbe and Colleagues 2016 (genetic testing costs) [10]
Medications	HSE PCRS*
Walking and other aids	Procurement Sourcing Department, TUH*
Home modifications	• Discharge planning team, TUH • Budget 2019, Report on Tax Expenditures [11].
Long-term care	Department of Public Expenditure and reform, Nursing Home Support Scheme: Tends and Figures, 2017 [12]
Respite or day care	• Ataxia Ireland • Department of Health, Value for Money and Policy Review of Disability Services in Ireland, 2012 [13]
Professional paid care	HSE National Service Plan, 2018 [14]
Transport	Transport for Ireland
Productivity/informal care	CSO, Earnings and labour costs [15]

Table 1 references: [9, 10, 16–22]

*GP* general practitioner, *HSE* health service executive, *ED* emergency department, *PCRS* Primary Care Reimbursement Service, *HIPE* Hospital Inpatient Enquiry, *TUH* Tallaght University Hospital, *CSO* Central Statistics Office

*Estimates were made from publicly available pricelists for select items on which reimbursement data was not available

General Practitioner (GP) visit costs for participants with medical cards were based on capitation rates, formed on age and gender, and not frequency of visits [10]. For patients without medical cards, the national average cost per visit was used.

Medication use was recorded according to medication class (e.g. muscle relaxant, medication for cholesterol). Costs of medication classes were calculated based on clinical judgement, taking conservative estimates on the most commonly prescribed and cheapest medication likely to be used in each class, assigning generic prices where possible.

Costs of genetic testing, where the exact test was not specified, were based on common clinical practice. FRDA and Non-FRDA participants (i.e. those with a known genotype) were assumed to have been tested for that specific gene. Participants in the ‘Unknown’ group were assumed to have undergone initial screening tests for the most common ataxias (FRDA, SCA1, 2, 3, 6 and 7) [9].

OHP visit costs were taken from the median point on relevant salary scales [16], assuming one visit to cost an hour’s wage. Chiropractor costs were based on the mean cost from eight clinics with publicly available price lists.

The costs of walking aids and medical devices were annualised assuming a 5-year lifespan and a 4% discount rate [11].

The cost of car modifications was based on the average value added tax refund claim on the disabled driver and passenger scheme [17].

The cost of long-term care facility residence was based on the average national expenditure on care home beds in 2016 [14].

Productivity losses were calculated using a human capital approach. Hours lost as a result of absenteeism or inability to work due to ataxia over the previous week was costed according to the national gross mean hourly wage in Ireland. Those forced to retire due to ataxia were also assumed to incur 40 hours a week of productivity losses unless they were over the retirement age of 65. Informal care costs were calculated similarly, based on the number of hours of care provided, capped at 40 hours per week. The age of caregivers was not recorded so all were assumed to incur indirect costs.

The hourly cost of professional care was based on national budget projections [18]. If care was received from both professional and informal caregivers, a median of the two hourly costs was applied as data was not available on the breakdown of care provision. The maximum cost of homecare was capped at €75,000 per annum, the maximum reimbursement allowed by the state. This avoided overestimation of the costs incurred by those requiring 24-h care.

Unit costs were inflated to 2016 prices where necessary, using the consumer price index available through the Central Statistics Office of Ireland.

### Cost-of-Illness Calculation

The mean COI for all participants was calculated using a prevalence-based approach and bottom-up methodology, i.e. by first measuring and quantifying the utilisation of individual services over a 12-month period, then multiplying these quantities by the associated unit costs for each participant [23]. If a frequency was expressed over a range (e.g. two to three times), the median of that range was used for the cost calculation (i.e. 2.5).

For recall periods less than a year, costs were annualised on the assumption that requirements were similar throughout the remaining year.

### Distribution of Costs

Costs were distributed between public (i.e. paid for by the state) and private (paid for by the participants) funding. Participants with medical cards, a means-tested support which covers costs of medical care, were assumed to have free access to all medical services and medications. Those without medical cards were assumed to have paid contributions towards inpatient hospital stays, emergency department (ED) visits and medications. Participants reported whether their home modifications were funded privately or publicly. Long-term care facility costs were assumed to be publicly funded as these costs could not be accurately distributed according to means testing. Transport costs to clinic were assumed to be funded privately. Indirect costs were assumed to be funded privately, minus any financial benefits (invalidity pension, disability allowance, domiciliary care allowance, mobility allowance and carer benefit) which were costed according to 2016 rates [24]. Other benefits, including old-age pension and fuel and rent allowances, were not costed as rates vary and are not directly related to disability or healthcare.

### Analysis

Statistical analysis was performed using IBM® SPSS® version 25. The data was checked and cleaned. Descriptive statistics regarding frequency of service usage are presented using means or medians and 95% confidence intervals. Mean costs are presented for individual cost-unit categories and for overall direct (medical and non-medical), indirect and total costs. 95% confidence intervals for mean costs were calculated using non-parametric bootstrapping due to the skewed nature of data distribution.

Demographic differences between ataxia subgroups and differences in cost according to selected demographic predictors were assessed for significance at the 0.05 level using appropriate statistical tests and post hoc analyses.

Missing data was excluded unless it could reasonably be inferred for the purposes of a more accurate cost analysis. Nine of 36 hospital stays did not have a documented length of stay (LOS). Therefore, these cases were assigned the average LOS of other visits. Three participants did not report the number of hours per day that they received homecare. They were assumed to have received 1 hour per day.

### Sensitivity Analyses

The following sensitivity analyses were performed on variables in which there was felt to be a significant degree of uncertainty:

1. Costs of informal or combined informal-professional care were reduced by 20.2%, the proportion of registered informal carers nationwide greater than 65 years of age in 2016 [25].
2. All informal care hours were assumed to be 26.15 hour per week, the average number of paid work hours lost by informal carers to FRDA patients in a similar UK study [4]. Patients receiving over 26.15 hour of combined care were assumed to receive professional care for the remaining hours.
3. The €75,000 cap to care costs was removed.
4. Each hospital inpatient stay was costed at €6841, the national average in 2016 [26], rather than according to length of stay in an illustrative hospital.
5. Home and car modification costs were varied by 25%.

### Ethical Approval

Ethical approval was obtained from the Tallaght University Hospital/St. James’ Hospital Joint Research Ethics Committee.

## Results

### Demographics

One hundred thirty-four surveys were returned, 85 on paper and 49 online. Five who reported acquired ataxia were excluded. Of the remaining 129, 56 (43%) had FRDA and 18 (14%) had a different type of inherited ataxia (Non-FRDA): these included ataxia-telangiectasia (*n* = 4), ataxia with oculomotor ataxia type 1 (*n* = 2), X-linked tremor/ataxia syndrome (FXTAS) (*n* = 2), episodic ataxia (*n* = 1), hereditary spastic paraplegia (*n* = 5; including *SPG7*-associated spastic ataxia (2), *SPG54*-spastic ataxia (2) and 1 unspecified), SCA (*n* = 3; including individuals with SCA2, SCA6 and SCA14) and SCAR10 (*n* = 1). Of the 129, 55 were classified as Unknown.

Key demographics are shown in Table 2. Participants with FRDA had an earlier age of onset (*p* < 0.001, Kruskal–Wallis analysis), were younger (*p* = 0.031) and more likely to be wheelchair-bound (*p* < 0.001) compared to the Unknown group.

**Table 2:** Baseline Demographics

			FRDA (n=56)		Non-FRDA (n=18)		Unknown (n=55)		All (n=129)	
			n	(%)	n	(%)	n	(%)	n	(%)
	Gender	Female	30	(53.6%)	8	(44.4%)	32	(58.2%)	32	(58.2%)
	Age (years)	<20	6	(10.7%)	1	(5.6%)	7	(12.7%)	14	(10.9%)
		20-39	28	(50.0%)	4	(22.2%)	7	(12.7%)	39	(30.2%)
		40-59	15	(26.8%)	8	(44.4%)	16	(29.1%)	39	(30.2%)
		≥60	7	(12.5%)	5	(27.8%)	25	(45.5%)	37	(28.7%)
	Age of symptom onset (years)	<20	46	(82.1%)	9	(50.0%)	22/53*	(41.5%)	77/127*	(60.6%)
		20-45	7	(12.5%)	5	(27.8%)	16/53*	(30.2%)	28/127*	(22.0%)
		>45	3	(5.4%)	4	(22.2%)	15/53*	(28.3%)	22/127*	(17.3%)
	Disease Duration (years)	<10	14	(25.0%)	6	(33.3%)	19/54*	(35.2%)	39/128*	(30.5%)
		10-19	19	(33.9%)	4	(22.2%)	13/54*	(24.1%)	36/128*	(28.1%)
		≥20	23	(41.1%)	8	(44.4%)	22/54*	(40.7%)	53/128*	(41.4%)
Occupational Status	Employed (full and part-time)		8	(14.3%)	0	(0.0%)	4	(7.3%)	12	(9.3%)
	Homemaker		3	(5.4%)	0	(0.0%)	4	(7.3%)	7	(5.4%)
	In education (primary or secondary)		3	(5.4%)	1	(5.6%)	7	(12.7%)	11	(8.5%)
	In education (higher level)		9	(16.1%)	1	(5.6%)	1	(1.8%)	11	(8.5%)
	Unable to work or retired due to Ataxia		26	(46.4%)	12	(66.7%)	23	(41.8%)	61	(47.3%)
	Retired due to age		1	(1.8%)^†^	2	(11.1%)	8	(14.5%)^†^	11	(8.5%)
	Other occupational situation		6	(10.7%)	2	(11.1%)	8	(14.5%)	16	(12.4%)
Living Circumstances	Living alone (+/- children)		10	(17.9%)	3	(16.7%)	9	(16.4%)	22	(17.1%)
	Living with spouse/partner(+/- children)		9	(16.1%)^†‡^	9	(50.0%)^†^	23	(41.8%)^‡^	41	(31.8%)
	Living with parents or other relatives		32	(57.1%)^†^	5	(27.8%)	17	(30.9%)^†^	54	(41.9%)
	Live in a care home / long-term facility		2	(3.6%)	1	(5.6%)	5	(9.1%)	8	(6.2%)
	Other living situation		3	(5.4%)	0	(0.0%)	1	(1.8%)	4	(3.1%)
Wheelchair use	Never		9	(16.1%)^†^	6	(33.3%)	25/54*	(46.3%)^†^	40/128*	(31.3%)
	Part-time		14	(25.0%)	4	(22.2%)	19/54*	(35.2%)	37/128*	(28.9%)
	Full-time		33	(58.9%)^†^	8	(44.4%)	10/54*	(18.5%)^†^	51/128*	(39.8%)

FRDA; Friedreich’s Ataxia

*Valid n specified due to missing data. Proportions are otherwise calculated from total n for each subgroup

^†^Identifies two figures in one row that differ significantly (p<0.05, Kruskal-Wallis analysis)

^‡^Identifies two figures in one row that differ significantly (p<0.05, Kruskal-Wallis analysis)

### Resource Utilisation

Table 3 illustrates the breakdown of resource utilisation.

**Table 3:** Resource utilisation

			FRDA (n=56)		Non-FRDA (n=18)		Unknown (n=55)		All (n=129)	
Healthcare encounters	Clinic visits	n (%)	50	(89.3%)	14	(77.8%)	45/54**	(83.3%)	109/128**	(85.2%)
		Mean number of visits (95% CI)	5.91	(3.69, 8.13)	4.18	(0.79, 7.57)	3.61	(2.51, 4.71)	4.74	(3.56, 5.92)
	GP visits	n (%)	37	(66.1%)	15	(83.3%)	37	(67.3%)	89	(69.0%)
		Mean number of visits (95% CI)	3.47	(2.36, 4.58)	6.2	(-0.85, 13.25)	4.23	(3.19, 5.27)	4.25	(3, 5.5)
	ED visits	n (%)	14	(25.0%)	3	(16.7%)	11	(20.0%)	28	(21.7%)
		Mean number of visits (95% CI)	1.43	(0.89, 1.97)	1	(1, 1)	1.27	(0.84, 1.71)	1.32	(1.02, 1.62)
	OHP visits	n (%)	47	(83.9%)	15	(83.3%)	48	(87.3%)	110	(85.3%)
		Median number of visits (95% CI)	8.5	(6, 13)	14.5	(6, 57)	7.25	(5, 11)	8.5	(6, 11.5)
	Inpatient stays*	n (%)	17	(30.4%)	4	(22.2%)	15	(27.3%)	36	(27.9%)
		Mean number of stays (95% CI)	1.65	(1.17, 2.13)	2.25	(-0.14, 4.64)	1.2	(0.89, 1.51)	1.53	(1.22, 1.84)
		Mean cumulative LOS	12.94	(4.32, 21.56)	29.67	(-46.51, 105.84)	9.9	(2.58, 17.22)	13.62	(7.33, 19.92)
			n	(%)	n	(%)	n	(%)	n	(%)
Investigations	Blood tests		31	(55.4%)	6	(33.3%)	22	(40.0%)	59	(45.7%)
	Genetic testing		2	(3.6%)^†‡^	4	(22.2%)^†^	15	(27.3%)^‡^	21	(16.3%)
	MRI		4	(7.1%)	2	(11.1%)	8	(14.5%)	14	(10.9%)
	CT		5	(8.9%)	0	(0.0%)	3	(5.5%)	8	(6.2%)
	X-ray		14	(25.0%)	1	(5.6%)	1	(1.8%)	16	(12.4%)
	Echocardiogram		25	(44.6%)^†‡^	2	(11.1%)^†^	3	(5.5%)^‡^	30	(23.3%)
	ECG/Holter		29	(51.8%)^†^	3	(16.7%)	5	(9.1%)^†^	37	(28.7%)
	Neurophysiology		5	(8.9%)	3	(16.7%)	4	(7.3%)	12	(9.3%)
Medications	Muscle relaxants		17	(30.4%)	7	(38.9%)	11	(20.0%)	35	(27.1%)
	Neuropathic painkillers: Pregabalin or Gabapentin		8	(14.3%)	3	(16.7%)	7	(12.7%)	18	(14.0%)
	Diabetes medication		12	(21.4%)	1	(5.6%)	5	(9.1%)	18	(14.0%)
	Anti-epileptic medication		0	(0.0%)	1	(5.6%)	3	(5.5%)	4	(3.1%)
	Cardiac medications (including antihypertensives)		22	(39.3%)^†‡^	1	(5.6%)^†^	9	(16.4%)^‡^	32	(24.8%)
	Cholesterol medication		12	(21.4%)	3	(16.7%)	10	(18.2%)	25	(19.4%)
	Antibiotics		7	(12.5%)	0	(0.0%)	5	(9.1%)	12	(9.3%)
	Painkillers/ NSAID		11	(19.6%)	1	(5.6%)	10	(18.2%)	22	(17.1%)
	Contraceptives		5	(8.9%)	0	(0.0%)	2	(3.6%)	7	(5.4%)
	Antidepressants		11	(19.6%)	2	(11.1%)	11	(20.0%)	24	(18.6%)
	Idebenone or Nicotinamide		2	(3.6%)	0	(0.0%)	1	(1.8%)	3	(2.3%)
	Co-enzyme Q10		14	(25.0%)	3	(16.7%)	3	(5.5%)	20	(15.5%)
	Vitamin E		10	(17.9%)^†^	0	(0.0%)^†^	2	(3.6%)	12	(9.3%)
	Omega 3 fatty acids		10	(17.9%)	2	(11.1%)	9	(16.4%)	21	(16.3%)
	Nutritional/vitamin supplements		18	(32.1%)	6	(33.3%)	17	(30.9%)	41	(31.8%)
Walking Aids	Walking-stick or crutches		8	(14.3%)	5	(27.8%)	10	(18.2%)	23	(17.8%)
	Frame (including rollator frame)		15	(26.8%)	3	(16.7%)	22	(40.0%)	40	(31.0%)
	Manual wheelchair		37	(66.1%)^†‡^	11	(61.1%)^†^	14	(25.5%)^‡^	62	(48.1%)
	Power wheelchair		19	(33.9%)^†^	5	(27.8%)	7	(12.7%)^†^	31	(24.0%)
	Power scooter		5	(8.9%)	1	(5.6%)	4	(7.3%)	10	(7.80%)
Medical Devices	Orthotics		25	(44.6%)^†^	5	(27.8%)	7	(12.7%)^†^	37	(28.70%)
	Back brace		2	(3.6%)	1	(5.6%)	0	(0.0%)	3	(2.30%)
	Hearing-aid		5	(8.9%)	0	(0.0%)	2	(3.6%)	7	(5.40%)
	Non-invasive ventilation		4	(7.1%)	0	(0.0%)	1	(1.8%)	5	(3.90%)
	Pacemaker/ICD		2	(3.6%)	0	(0.0%)	3	(5.5%)	5	(3.90%)
	Insulin pump		1	(1.8%)	0	(0.0%)	0	(0.0%)	1	(0.80%)
	Urinary catheter		5	(8.9%)	0	(0.0%)	1	(1.8%)	6	(4.70%)
Home modifications			32	(57.1%)	13	(72.2%)	26	(47.3%)	71	(55.0%)
Care	Professional care		15	(26.8%)	7	(38.9%)	15	(27.3%)	37	(28.7%)
	Informal care		13	(23.2%)	5	(27.8%)	18	(32.7%)	36	(27.9%)
	Both		12	(21.4%)	2	(11.1%)	4	(7.3%)	18	(14.0%)
	Total		40	(71.4%)	14	(77.8%)	37	(67.3%)	91	(70.5%)
Out-patient Care	Attended respite		14	(25.0%)	5	(27.8%)	13	(23.6%)	32	(24.8%)
	Attends Day Centre		8	(14.3%)	4	(22.2%)	8	(14.5%)	20	(15.5%)
	Transport to clinic		25	(44.6%)	9	(50.0%)	19	(34.5%)	53	(41.1%)
Health-care Cards	Medical card		40	(71.4%)	14	(77.8%)	39	(70.9%)	93	(72.1%)
	LTI card		2	(3.6%)	0	(0.0%)	2	(3.6%)	4	(3.1%)
	Both		8	(14.3%)	0	(0.0%)	2	(3.6%)	10	(7.8%)
Financial support	Invalidity Pension		4	(7.1%)	4	(22.2%)	9	(16.4%)	17	(13.2%)
	Disability Allowance		31	(55.4%)^†‡^	6	(33.3%)^†^	9	(16.4%)^‡^	46	(35.7%)
	Domicilliary Care Allowance		1	(1.8%)	1	(5.6%)	3	(5.5%)	5	(3.9%)
	Mobility Allowance		3	(5.4%)	0	(0.0%)	0	(0.0%)	3	(2.3%)
	Carer benefit		18	(32.1%)	3	(16.7%)	13	(23.6%)	34	(26.4%)
	Other/Not specified		7	(12.5%)	4	(22.2%)	7	(12.7%)	18	(14.0%)

Hospital clinic visits included a range of specialities: Paediatrics, neurology, cardiology, respiratory, orthopaedics, urology, endocrinology, psychiatry, ophthalmology, pain medicine, elderly, care, general surgery and neurosurgery

Home modifications included adaptations such as accessibility ramps and rails, renovation of the bathroom, kitchen or entire home and car adaptations

CI; Confidence Interval, FRDA; Friedreich’s Ataxia, GP; General Practitioner, ED; Emergency Department, OHP; Other Healthcare Professionals, MRI; Magnetic Resonance Imaging, CT; Computed Tomography, ECG; Electrocardiogram, NSAID; Non-Steroidal Anti-Inflammatory Drug, ICD; Implantable Cardiac Defibrillator, LTI; Long-Term Illness

*Note that data for LOS was missing for 9 admissions. Mean is calculated from available data

**Valid N specified due to excluded data. Proportions are otherwise calculated from total n for each subgroup

^†^Identifies two figures in one row that are differ significantly (p<0.05, chi-square analysis)

^‡^Identifies two figures in one row that are differ significantly (p<0.05, chi-square analysis)

Of the 129, 109 (84.5%) participants attended a hospital consultant clinic an average of 4.74 times. The specialty attended by the greatest number of participants was neurology (*n* = 95, 73.6%; mean number of visits = 1.81), followed by cardiology (*n* = 44, 34.1%; mean number of visits = 1.45) and ophthalmology (*n* = 37, 28.7%; mean number of visits = 1.92). OHP visits over the previous year are expressed as a median (8.5; 95% CI 6, 11.5; *n* = 110) due to a small number of right-sided outliers (mean = 20.15; 95% CI 14.54, 25.77). The most commonly attended OHP was PT (*n* = 76, 58.9%; mean number of visits = 12.08), followed by OT (*n* = 73, 56.6%; mean number of visits = 4.26) and SALT (*n* = 45, 34.9%; mean number of visits = 4.08). These results were similar for the FRDA group: PT (*n* = 33, 58.9%, mean number of visits = 13.05), OT (*n* = 35, 62.5%, mean number of visits = 3.93) and SALT (*n* = 19, 33.9%, mean number of visits 1.95).

### Cost of Illness

The breakdown of costs is illustrated in Table 4. The average total COI was €60,020 per person per year (PPPY).

**Table 4:** Sources of unit costs

		FRDA (n=56)			Non-FRDA (n=18)			Unknown (n=55)			All (n=129)		
Direct medical costs		n	Mean cost (€)	95% CI	n	Mean cost (€)	95% CI	n	Mean cost (€)	95% CI	n	Mean cost (€)	95% CI
	GP Visits	54	89^†^	(80, 100)	18	111	(90, 132)	51	140^†^	(120, 163)	123	113	(102, 125)
	Outpatient Clinic Visits	50	809	(615, 1,059)	14	638	(282, 1,154)	46	527	(408, 663)	110	669	(539, 797)
	ED visits and inpatient stays	19	8,956	(4,050, 15,350)	5	19,409	(3,768, 39,536)*	19	5,973	(3,377, 9,228)	43	8,854	(5,658, 13,138)
	OHP visits	47	420	(286, 593)	15	1,174	(451, 2,071)	48	340	(236, 469)	110	488	(357, 653)
	Investigations(6 months)	45	1,296	(937, 1,688)	8	1,107	(445, 1,838)	30	932	(557, 1,355)	83	1,146	(878, 1,414)
	Medications	47	183	(141, 233)	13	182	(108, 264)	38	161	(108, 216)	98	174	(143, 206)
Total direct medical costs		42	5,873	(4,002, 8,151)	16	8,124	(1,713, 17,856)	51	4,376	(3,203, 5,840)	109	5,508	(4,086, 7,126)
Direct non-medical costs	Long-term care facility	2	54,963	-	1	54,963	-	5	54,963	-	8	54,963	-
	Cost respite or day care	17	5,916	(4,095, 7,850)	6	6,163	(3,999, 7,997)	17	5,329	(3,391, 7,376)	40	5,703	(4,610, 6,934)
	Transport	25	328	(262, 389)	9	390	(277, 468)*	19	382	(316, 439)	53	358	(313, 396)
	Professional care	15	33,079	(20,213, 47,701)	7	13,123	(8,557, 18,512)	15	17,550	(7,520, 31,811)	37	23,008	(16,152, 31,771)
	Walking and other aids	50	378^†^	(240, 585)	16	153	(73, 247)	41	230	(103, 401)	107	288	(200, 393)
	Home Modifications	32	15,820	(10,278, 21,728)	13	18,366	(8,659, 30,078)	26	13,840	(7,092, 22,344)	71^†^	15,561	(11,208, 19,686)
Total direct medical costs		42	5,873	(4,002, 8,151)	16	8,124	(1,713, 17,856)	51	4,376	(3,203, 5,840)	109	5,508	(4,086, 7,126)
Direct non-medical costs	Long-term care facility	2	54,963	-	1	54,963	-	5	54,963	-	8	54,963	-
	Cost respite or day care	17	5,916	(4,095, 7,850)	6	6,163	(3,999, 7,997)	17	5,329	(3,391, 7,376)	40	5,703	(4,610, 6,934)
	Transport	25	328	(262, 389)	9	390	(277, 468)*	19	382	(316, 439)	53	358	(313, 396)
	Professional care	15	33,079	(20,213, 47,701)	7	13,123	(8,557, 18,512)	15	17,550	(7,520, 31,811)	37	23,008	(16,152, 31,771)
	Walking and other aids	50	378^†^	(240, 585)	16	153	(73, 247)	41	230	(103, 401)	107	288	(200, 393)
	Home Modifications	32	15,820	(10,278, 21,728)	13	18,366	(8,659, 30,078)	26	13,840	(7,092, 22,344)	71^†^	15,561	(11,208, 19,686)
Total direct non-medical costs		39	29,307	(18,040, 40,988)	15	21,317	(10,201, 32,574)	45	32,574	(12,260, 32,822)	99	24,342	(18,490, 31,896)
Total direct costs		42	33,087	(22,179, 46,067)	16	28,109	(15,332, 41,176)	51	22,959	(14,601, 32,774)	109	27,617	(21,255, 34,468)
Proportion funded publicly		81.5%			85.7%			77.8%			80.7%		
Indirect costs	Informal Care	13	27,374	(17,632, 37,545)	5	37,133	(16,809, 45,843)*	18	40,113	(34,108, 44,805)	36	35,099	(29,798, 40,080)
	Productivity	25	44,147	(40,194, 45,843)	11	45,843	(45,843, 45,843)	18	43,551	(38,342, 45,843)	54	44,294	(41,753, 45,843)
Total Indirect costs		25	45,408	(37,101, 54,152)	11	54,387	(45,843, 66,216)	30	50,198	(41,954, 58,818)	66	49,082	(43,733, 54,831)
Proportion funded publicly		34.7%			22.5%			20.2%			25.7%		
Total costs		56	62,607	(50,914, 76,248)	18	74,177	(54,094, 95,389)	55	52,752	(43,112, 63,686)	129	60,020	(52,931, 67,214)
Proportion funded publicly		60.5%			49.6%			45.4%			52.2%		

Costs are broken down into direct and indirect costs. Direct costs are further broken down into medical and non-medical costs. The proportion of direct, indirect and total costs funded by publicly (i.e. by the state, health service or local services) is also displayed

95% CIs are estimated using a bootstrapping technique with 1000 samples, unless otherwise stated. The annual costs per person of long-term care facility reference was based on a single national average, therefore 95% CIs are not reported

CI; Confidence Interval, FRDA; Friedreich’s Ataxia, GP; General Practitioner, ED; Emergency Department, OHP; Other Healthcare Professionals

*<1000 bootstrapping samples

^†^Identifies two figures in one row that are differ significantly (p<0.05, Kruskal-Wallis analysis)

Costs are divided into direct (medical and non-medical) and indirect. Only one participant incurred no costs over the 12-month period.

Eighteen participants reported receiving both professional and informal care and one participant did not specify the caregiver types. Cost of home care could therefore not be distributed as direct or indirect for these 19 cases and they were excluded from indirect and direct cost subtotals. All cases were included in the final COI calculation as this included all direct and indirect costs.

Indirect costs were significantly higher than direct costs (*p* < 0.001, Wilcoxon signed-rank test) and accounted for 51.9% of total costs. Of the 54 (41.86%) individuals under the retirement age who reported missing work hours due to illness, all but 2 (i.e. 52, 40.3%) reported being unable to work at all due to ataxia.

The cost of informal care could be considered an overestimation, as it is not known for certain whether caregivers would otherwise have spent these hours in paid employment, or whether they were retired due to age. Attempts to account for the latter were included in the sensitivity analysis.

There were no statistically significant differences in direct, indirect or total costs between subgroups. Statistically significant differences in individual costs are highlighted in Table.

Table 5 illustrates associations between demographic variables and cost. Differences in disease duration, occupational status, living circumstances and wheelchair use were all associated with statistically significant differences in cost. Ataxia type, gender, current age and age at disease onset were not.

**Table 5:** Differences in Cost according to demographic variables

	52,628 (33,808, 71,449)	N	Mean Cost, € (95% CI)	P*
Ataxia Type	FRDA	56	62,607 (50,423, 74,792)	0.245
	Non-FRDA	18	74,177 (52,041, 96,313)	
	Unknown	55	52,752 (42,232, 63,271)	
Gender	Female	70	61,297 (50,623, 71,971)	0.702
	Male	59	58,504 (47,811, 69,198)	
Age (years)	</= 20 Years	14	44,663 (23,467, 65,860)	0.192
	20-64 years	92	64,204 (55,232, 73,177)	
	65+ years	23		
Age of symptom onset (years)	<20 years	77	59,023 (49,031, 69,015)	0.8
	21-45 years	28	72,717 (55,447, 89,987)	
	>45 years	22	45,610 (30,521, 60,699)	
Disease Duration (years)	</=10 years	39	47,438 (34,190, 60,687)	0.15
	11-20 years	36	55,679 (41,423, 69,935)	
	>20 years	53	71,418 (59,637, 83,200)	
Occupational Status	Other occupational status	68	39,649 (30,350, 48,948)	<0.001
	Unable to work or had to retire due to Ataxia	61	82,728 (73,516, 91,940)	
Living Circumstances	Lives alone or with spouse/partner (+/- children)	63	57,547 (47,934, 67,160)	0.003
	Lives with parents or other relatives	54	55,247 (44,215, 66,279)	
	Lives in a long-term care facility	8	115,255 (81,634, 148,876)	
Wheelchair use	No	40	51,006 (36,932, 65,080)	0.038
	Yes	88	64,594 (55,686, 73,503)	

*Mean costs compares using Mann-Whitney U and Kruskall-Wallis tests as appropriate

### Cost Distribution

Costs were distributed between public and private funding. The cumulative publicly funded costs are expressed in Table 4. Of the direct, indirect and total costs, 80.7%, 25.7% and 52.2% respectively were publicly funded.

The claiming of various financial support benefits is illustrated in Table 3. It should be noted that 7 of the 34 participants reporting receipt of carer benefit reported receiving professional care only (not informal care) and 8 reported no homecare. This disparity may be explained by the phrasing of questioning in the survey; participants were asked (on the basis of the pilot study) whether they received any informal care provided *free* by family/friends and thus may have not have reported care from those receiving carer benefit.

### Sensitivity Analysis

The results of sensitivity analyses are illustrated in Table 6. All but one analysis found a significantly different outcome from the base case. Mean total costs in sensitivity analysis ranged from €57,797 to €67,772.

**Table 6:** Sensitivity Analysis

		Mean Cost, € (95% CI)	% Change	P*
Base Case		60,020 (52,536, 67,503)	-	-
Costs of informal care reduced by 20.2%**		57,797 (50,436, 65,158)	-3.7	<0.001
Informal care requirements 26.15 hours/week		59,977 (52,824, 67,130)	3.6	0.926
No upper limit on care costs		67,772 (56,909, 78,635)	13.0	0.005
Hospital stays costed according to national average		61,940 (54,171, 69,710)	-9.7	0.019
Home and car modifications	↓ 25%	57,879 (50,636, 65,121)	-6.8	<0.001
	↑ 25%	62,646 (54,870, 70,423)	7.9	<0.001

Mean total cost per patient from five separate sensitivity analyses are compared to the base case

**20.2% of registered informal carers in Ireland in 2016 were over 65 years of age

*Wilcoxon signed rank test

CI; Confidence intervals

## Discussion

This study is the first to attempt to calculate the COI of inherited ataxia in Ireland. Furthermore, it is, to our knowledge, the largest single-country ‘real-life’ patient survey in Europe to evaluate patients with different inherited ataxia subtypes. Our results demonstrate the significant financial burden of inherited ataxia in Ireland and highlight the considerable indirect costs. Comparison of these results with those in other countries [2, 3] and other neurological conditions in Ireland [27–30] illustrates the financial burden these conditions place on people with ataxia, their caregivers, the healthcare system and society as a whole.

### Overview of Inherited Ataxia in Ireland

The prevalence of inherited ataxia in Ireland is unknown. When this study was conducted, Ataxia Ireland had 267 registered members, some of whom were family members rather than people with ataxia and others had acquired ataxia, and therefore, while a risk of selection bias exists, this high response rate likely enhances the accuracy of our demographic data as a representation of adults with inherited ataxia throughout Ireland.

FRDA participants were more likely to live with their parents and less likely to live with a spouse. This may reflect age differences attributed to the fact that FRDA typically presents in early life [27]. However, individuals with FRDA were also more likely to require a wheelchair, suggesting a greater level of disability and dependence, possibly necessitating this co-habitation with family members.

### Resource Utilisation

People with inherited ataxia require comprehensive healthcare input, as demonstrated by our results. Furthermore, it is likely that the true clinical need is actually greater than reported here but is not being met by the health service. It is recommended that individuals with FRDA have annual follow-up with a neurologist [28], cardiac assessment with echocardiogram and ECG and a blood test for diabetes [29]. However, 26.4% of FRDA participants had not been reviewed by a neurologist and 55% did not have an echocardiogram in the year prior to survey. Similarly, 41% and 66% of FRDA participants did not have PT and SALT input respectively, even though it is recommended that they receive regular input from these specialties [29].

The reason for not meeting such standards is likely to be multifactorial, including patient concordance and preference as well as emerging delays in clinic assessments due to increasing demand and limited resources. The Republic of Ireland has one of the lowest ratios of neurologists to population in the developed world; 1/132,352 compared to a median of 4.84/100,000 across Europe [30]. It is likely that the COI would be even higher if the recommended standards of care were achieved.

FRDA participants were more likely to take cardiac medications and vitamin E, undergo cardiac investigations and be on disability benefit, reflecting the typical phenotype and management strategies in FRDA [29]. They were less likely to have had genetic testing in the past year as the genetic diagnosis tends to be made relatively early in the disease course.

The results of this study suggest that a diagnosis of inherited ataxia confers high levels of disability and dependence. Over two thirds of participants required professional or informal homecare and used a wheelchair. Disability associated with inherited ataxia is incapacitating; almost half of participants reported being unable to work or retiring due to their illness. This level of disability increases with time as the disease progresses, impacting on quality of life and incurring significant direct and indirect costs.

### Cost of Illness

The average COI for inherited ataxia in our study was approximately €60,000 PPPY. If this is extrapolated to all members of Ataxia Ireland in 2016 (though this is unlikely to include all people with inherited ataxia in Ireland), the COI would be greater than €16 million.

Longer disease duration, inability to work due to ataxia, long-term care facility residence and wheelchair use were all associated with greater costs, suggesting that costs increase as disease progresses, and disability levels increase.

Comparison of results with other studies is limited by differences in study technique. The *direct* COI for a FRDA patient in North America in 2010 was reported as 14,144 USD (€12,730, all comparative costs have been inflated to 2016 prices and converted using the average 2016 exchange rate) in the USA and 38,373 CAD (€26,104) PPPY in Canada [3]. The latter figure is similar to the direct costs in our study. The USA figure cannot be accurately compared as it did not include the costs of long-term care facilities, medical devices and home or car modifications. A study of similar design to ours conducted in the UK, but again only including FRDA patients [4] estimated the COI to be £23,110 (€28,183) PPPY, almost half of our estimate. The rates of resource utilisation are roughly equivalent between the two studies, though participants in our study reported higher rates of respite care and home modifications. While this disparity could imply higher unit costs in Ireland, it may also reflect differences in methodology (such as productivity loss calculation) and the inclusion of additional variables in our study such as long-term care facility residence, car modifications and medical devices [4].

Indirect costs accounted for 52% of total COI. This is in keeping with other neurological disorder COI studies which demonstrate that indirect costs calculated using a human capital approach have a more significant financial impact on society that direct costs [4, 31, 32].

In Ireland, inherited ataxia is costly compared with other neurological illnesses. The COI is greater than that of patients with mild or moderate severity multiple sclerosis (MS) (€45,482), but less than severe MS (€97,318) [31]. This result is notable as inherited ataxia lacks the costly disease-modifying therapies available for people with MS. The higher costs amongst ataxia patients are primarily due to costs of home modifications and homecare, suggesting a greater level of physical disability. Dementia in Ireland has been calculated to cost €40,300 PPPY [32]. While this includes indirect costs, the resource utilisation was based on national figures rather than survey data and excludes home modifications. Direct costs of care in the first year after stroke [8] were lower (€20,143) in 2018 than annual direct costs in our study, though patients who died in hospital or were discharged to residential care facilities were not included in the latter study. The direct costs of amyotrophic lateral sclerosis were found to be similar (€21,552) but this only assessed costs paid by the health service and neither home modifications nor long-term care facility residence was reported [12].

### Cost Distribution

The costs of inherited ataxia place a high financial burden not only on the Irish healthcare system, but on patients and caregivers themselves. The majority of participants in our study held medical cards which entitled them to free GP and public hospital care. Eligibility for medical cards is based on household income, and while certain chronic illnesses qualify individuals for free medication and equipment related to their illness, ataxia does not confer such a qualification. Nineteen percent of direct costs were still paid privately by participants, and while this may be subsidised by private health insurance or support from charitable organisations, these will not eliminate costs entirely. Furthermore, this proportion is almost certainly an underestimate as public funding of long-term care facilities is only available on a means-tested basis. Some participants may also have acquired services privately due to long waiting lists and high demand in the public system.

While various financial supports from the state subsidise the indirect costs from productivity losses, almost 75% of these societal costs still fall to patients and caregivers. Improving employment rates and productivity amongst people with inherited ataxia and other neurological disabilities may help to relieve the financial burden on society and patients while also benefiting quality of life [13].

### Strengths and Limitations

A major strength of this study is the sample size. This is, in part, due to the inclusion of all subtypes of inherited ataxia in our cohort, reflecting the range of cases seen in specialist ataxia clinics. Nevertheless, our data selection methods do carry risks of selection bias. Recruitment from clinics and society meetings may be biased towards less disabled patients who are fit to travel. The online survey and option for carers to complete surveys on a participant’s behalf aimed to reduce this bias, but assumes internet access and active engagement in patient organisations. It is difficult to assess the extent of these biases as there are no published epidemiological studies of ataxia in Ireland.

Attaining a sample of this size requires an accessible method of data collection and our survey was designed with this in mind. However, the retrospective participant-reported nature of the survey without the use of medical records or face-to-face interview carries the limitation of recall bias and limits the complexity of questions that could reasonably be asked (e.g. names and doses of medications). The categorical nature of questioning may lead to inaccuracies in the data (for instance, when reporting frequencies and timescales).

Variable unit costs such as home and car modifications had to be based on best estimates and may be less accurate than individually reported expenses. Modification costs in our study are higher than but comparable to the average reimbursement on modifications for people with disabilities in 2016 (10,116 per person) [15]. This figure does not include car modifications and is likely to underestimate the true cost as grants were capped at €30,000 and means tested. Where necessary, the more conservative estimates of costs were used. For example, patients requiring genetic testing often undergo sequential testing for multiple genes or costly next-generation sequencing before a diagnosis is identified [9]. The cost of GP visits for medical card holders was calculated using national capitated rates, though patients with ataxia are likely to require more visits and resources than the general population therefore the true cost to the state is likely to be much higher.

Several assumptions of our study methods have been tested in sensitivity analysis. While reducing informal care costs to account for carers aged over 65 had an unsurprisingly significant effect on total cost, this cost estimate is likely to be at the very lower limit since the reduction was also applied to those receiving combined care. It could also be argued that caregivers who would otherwise be retired from employment should still incur costs when using a human capital as, in their absence, care may be needed to be provided by professional or employed informal caregivers.

Informal care requirements were similar to those reported by Giunti and colleagues [4] in the UK. An upper limit of €75,000 was applied to care costs, affecting 10 participants. This was a necessary measure as several participants required 24-h professional care which is likely to cost less per hour than intermittent care but an accurate estimation of this cost was not available. While taking the average national cost of an inpatient stay would have increased the total cost, our method, which is calculated according to length of stay with our hospital as an index, was felt to be more accurate.

National data is not broken down into daily costs and presumably also incorporates the costs of inpatient investigations which in our study were calculated separately [26]. So, while these assumptions have a statistically significant effect on the total cost, they each have a clear rationale and the lower limit cost of €55,000 from sensitivity analysis remains a substantial figure.

It is reasonable to assume that participants with a known diagnosis (i.e. FRDA and Non-FRDA groups) are accurately reporting their diagnoses, though there is a small risk that participants are mistaken. However, the group with unknown ataxia will contain a wide range of ataxia subtypes and may include cases of FRDA and more likely Non-FRDA, in whom the diagnosis has not yet been made or is not known to the patient. Furthermore, comparisons with the relatively small Non-FRDA group carry a risk of type II error. Thus, comparisons drawn between the subgroups of this study should be viewed with caution.

## Conclusion

Inherited ataxias carry high financial costs to the health system, patients, caregivers and society as a whole. Costs are similar between FRDA and other forms of inherited ataxia and grow as disability increases over the course of the illness. Indirect costs or ‘productivity losses’ make up half the COI and place a significant financial burden on patients. Despite this, there is evidence to suggest that certain clinical standards of care such as frequency of clinician review, OHP input and monitoring investigations are not being met, possibly reflecting the limited availability of resources. The results of this study advocate the need for greater funding in inherited ataxia care in Ireland to ease the financial burden on patients and improve resource availability.

## Data Availability

The datasets generated and analysed during the current study are available from the corresponding author on reasonable request.
